# Use of dipeptidyl peptidase-4 inhibitors was associated with a lower risk of Parkinson’s disease in diabetic patients

**DOI:** 10.1038/s41598-023-49870-z

**Published:** 2023-12-15

**Authors:** Yu-Hsuan Lin, Chih-Cheng Hsu, Jia-Sin Liu, Kuo-Cheng Chang, Jin-An Huang

**Affiliations:** 1 Division of Neurology, Neurological Institute, Taichung Veterans General Hospital, 1650 Taiwan Boulevard Sect. 4, Taichung, 40705 Taiwan; 2 Institute of Population Health Sciences, National Health Research Institutes, Zhunan, Miaoli, 35053 Taiwan; 3 National Center for Geriatrics and Welfare Research, National Health Research Institutes, Yunlin, Taiwan; 4 Department of Family Medicine, Min-Sheng General Hospital, Taoyuan, 33044 Taiwan; 5 Department of Health Business Administration, Hungkuang University, Taichung, 43302 Taiwan

**Keywords:** Parkinson's disease, Diabetes, Risk factors

## Abstract

Diabetes mellitus is a risk factor for Parkinson's disease (PD). While animal studies have supported the benefits of incretin-based therapies, including dipeptidyl peptidase-4 (DPP4) inhibitors, in PD, clinical research has yielded controversial results. This cohort study aimed to assess the relationship between PD incidence and the utilization of DPP4 inhibitor in diabetic patients. Using Taiwan's National Health Insurance Research Database from 2009 to 2018, diabetic patients receiving metformin plus at least one second-line oral antidiabetic (OAD) were enrolled. The patients were categorized as DPP4 inhibitor users and non-users. Propensity score matching was employed to establish a 1:1 ratio between DPP4 inhibitor users and non-users. Among the 205,910 patients enrolled, 149 were diagnosed with PD during follow-up. The incidence rate was 0.29 per 1000 person-years for DPP4 inhibitor users and 0.55 per 1000 person-years for the non-users. DPP4 inhibitor users exhibited a significantly lower risk of PD (adjusted hazard ratio, 0.51; 95% CI 0.39–0.68). Among DPP4 inhibitor users, vildagliptin showed the strongest correlation with a reduction in the risk of PD. This study demonstrates that the use of DPP4 inhibitors along with metformin in diabetic patients is associated with a lower risk of PD compared to those using other OADs.

## Introduction

Parkinson’s disease (PD) is the second most common neurodegenerative disease^1^. It is characterized by progressive degeneration of dopaminergic neurons in the substantia nigra pars compacta, leading to dopamine deficiency in the striatum^2^. Several pathophysiological mechanisms have been proposed, including the aggregation and deposition of misfolding α-synuclein in the neurons^3^, the prion-like propagation of pathological α-synuclein^4^, impaired mitophagy^5^, mitochondrial dysfunction, the accumulation of reactive oxidative species^6^, and neuroinflammation associated with microglia and astrocytes^7^. The management of PD focuses on alleviating both motor and non-motor symptoms. To date, attempts to develop disease-modifying agents for PD have yielded inconclusive evidence^8^.

Type 2 diabetes mellitus (T2DM) is a metabolic disease characterized by impaired glucose metabolism, resulting from insulin resistance or impaired insulin secretion^9^. Studies have shown that diabetic patients have a 38% increased risk of developing PD^10^. Emerging evidence suggests that incretin-based antidiabetic therapies have neuroprotective effects and the potential of disease modification for PD. Two clinical trials have demonstrated that Exenatide, a glucagon-like peptide-1 (GLP-1) receptor agonist, improves both cognitive symptoms and motor deficits of PD patients^11,12^. Dipeptidyl peptidase-4 (DPP4) inhibitors, another incretin-based antidiabetic therapy, lower blood sugar levels by preventing the degradation of GLP-1 and glucose-dependent insulinotropic polypeptide (GIP)^13^. Preclinical studies have revealed that DPP4 inhibitors restore impaired dopamine release in the nigrostriatal system^14^ and cognitive decline caused by neuronal insulin resistance^15^. Although omarigliptin can cross the blood–brain barrier (BBB) because of its low molecular weight and lipophilic property^13^, most DDP4 inhibitors have limited BBB penetration. DPP4 inhibitors exert their neuroprotective properties indirectly by increasing the expression of GLP-1 and GIP^16^.

To our knowledge, four population-based studies have explored the association between the risk of PD and the utilization of DPP4 inhibitors. A previous study by Svenningsson et al*.* found that DPP4 inhibitors were correlated with a decreased incidence of PD, although the sample size was small^17^. However, subsequent studies yielded conflicting results^18–20^. The purpose of this study was to investigate the association between DPP4 inhibitor therapy and the incidence of PD in diabetic patients within the Taiwanese population. We used Taiwan’s National Health Insurance Research Database to evaluate the effects of DPP4 inhibitors in combination with metformin compared to other oral antidiabetics (OADs) plus metformin on the risk of PD.

## Methods

### Data source

The National Health Insurance Research Database (NHIRD), which is administered by the National Health Insurance program, contains the medical records of more than 99% of Taiwan’s 23 million residents dating back to 1995. Hence, the data are representative of the Taiwanese population. This database comprises information regarding demographic characteristics, diagnostic codes, prescription drugs, and medical procedures. The disease diagnoses were defined based on the International Classification of Diseases, 9th Revision, Clinical Modification (ICD-9-CM) and 10th Revision, Clinical Modification (ICD-10), before and since 2016, respectively.

### Ethical statement

This study was approved by the Institutional Review Board of the National Health Research Institutes (NHRI, EC1060704-E). With regard to patient privacy, personal identifiable information was encrypted. Due to the retrospective nature of the study and anonymization of all data before analysis, the need of informed consent was waived by the Institutional Review Board of the NHRI. All methods were performed in accordance with the applicable guidelines and regulations.

### Study design and participants

We conducted a retrospective longitudinal cohort using the NHIRD to determine the correlation between PD and DPP4 inhibitors in the Taiwanese population. Because metformin is recommended as the initial antidiabetic agent according to the practice guideline^21^, and DPP4 inhibitors are often used as an add-on therapy, our study included newly-diagnosed diabetic patients with metformin as a first-line therapy between January 1, 2009, and December 31, 2018. The diabetic patients were identified as those with at least two outpatient visits or hospitalization with a diagnosis of T2DM. We excluded cases that met the following criteria: (1) medical records indicating the use of antidiabetic medication or a diagnosis of diabetes before January 2009; (2) prior diagnosis of PD (ICD-9-CM code: 332.0, ICD-10 code: G20), secondary parkinsonism (ICD-9-CM code: 332.1, ICD-10 code: G21) or dementia; (3) prescription of second-line OAD, including sulfonylureas, meglitinides, alpha-glucosidase inhibitors, and thiazolidinediones (TZDs) before January 2009; (4) lack of a minimum period of 90 days of prescription of second-line OAD; (5) prescription of two or more types of second-line OADs; (6) prescription of insulin; (7) age younger than 18 years old; and (8) insufficient or missing data.

The comorbidities, including head injury, hypertension, hyperlipidemia, chronic heart failure (CHF), cerebrovascular disease, chronic kidney disease (CKD), obesity, chronic obstructive pulmonary disease (COPD), and cancer, were identified based on at least one inpatient record or two outpatient visits within one year before the index date. Due to the insidious onset nature of diabetes, the exact onset date is indeterminable. Therefore, we have decided to use the date of the first diagnosis of T2DM as the basis for calculating the duration of diabetes. The duration of diabetes was calculated from the date of initial diagnosis to the index date. In addition, drug utilization, such as OADs, angiotensin conversion enzyme inhibitors (ACEi), angiotensin II receptor blockers (ARB), beta-blockers, calcium channel blockers (CCBs), statins, and non-steroidal anti-inflammatory drugs (NSAIDs), was determined by prescription for more than 28 tablets within one year before the index date. The ICD-9-CM and ICD-10 codes for the above comorbidities, as well as the list of medication prescriptions, are provided in Tables S1 and S2, respectively. The daily defined dose (DDD) for each drug was the assumed average maintenance dose per day based on the recommendations of the WHO International Working Group for Drug Statistics Methodology^22^. The total amount of DPP4 inhibitors in milligrams one year before the PD diagnosis was converted into DDDs, and the duration of DPP4 inhibitor usage (in days) was determined from the first to the last fills. The average DDD for each individual was calculated by dividing the total DDD in the year by the period of DPP4 inhibitor usage. The detailed equations of average DDD are presented below:

$$\begin{aligned} & Total \;usage \;amount \;in \;DDDs = \frac{{Total \;DPP4\; inhibitor \;dosage\; in \;the\; preceding\; year \;of\; PD \;diagnosis\; ({\text{mg}})}}{{DDD\; ({\text{mg}})}} \\ & Average \;DDD = \frac{Total \;drug \;usage \;amount\; in \;DDDs }{{Duration\; of \;DPP4 \;inhibitor \;usage\; ({\text{days}})}} \\ \end{aligned}$$

### Outcome variables: Parkinson’s disease

The index date was defined as the date of PD diagnosis. PD diagnosis was defined as records of at least one inpatient and two outpatient medical claims of Parkinson’s disease (ICD-9-CM code: 332.0, ICD-10 code: G20). To minimize the possibility of misdiagnosis, the patients needed to receive antiparkinsonian medication within 30 days after PD diagnosis, either levodopa, dopamine agonist, monoamine oxidase B inhibitor, or a combination of these medications. Additionally, the interval between the first and the last visits had to be longer than 90 days. In parallel, those with a diagnostic code of secondary parkinsonism (ICD-9-CM code: 332.1, ICD-10 code: G21.11, G21,19 and G21.8) were excluded^23^. The follow-up period began from the eligible date, which is the initial diagnosis date of T2DM between 2009 and 2018, and ended on whichever of the following occurs first: the index date, withdrawal from the NHIRD, or December 31, 2018.

### Statistical analysis

To achieve balance factors between the DPP4 inhibitor user and non-user groups in terms of age, sex, comorbidities, and prescriptions, we employed propensity score matching at a 1:1 ratio. Logistic regression was used to generate the propensity score, with DPP4 inhibitor usage as the dependent variable and age, sex, comorbidities, and prescriptions as independent variables. This method minimized differences between the two groups. Baseline demographics were compared using the independent t-test for continuous variables and χ^2^ test for categorical variables. The standard mean difference was used to assess differences between variables after the propensity score matching. We used the log–log plot to evaluate whether the variables in this model violated the assumption of proportional risk. Hazard ratios and 95% confidence interval for PD among different groups were calculated using multivariate Cox regression models. Crude ratio was computed in Model 1, while Model 2 was adjusted for several variables, including age, gender, diabetic duration, hypertension, hyperlipidemia, obesity, cerebrovascular disease, CKD, COPD, head injury, cancer, calcium channel blockers, ACEIs or ARBs, statins, and NSAIDs. For Model 3, we adjusted for the variables used in Model 2 after performing propensity score matching. The cumulative PD incidence was assessed using the Kaplan–Meier curve and log-rank tests. We further examined the dose-dependent effects of DPP4 inhibitors by comparing users with the dosage above and below the median value. Meanwhile, the sub-distribution of death-adjusted competing risk model was also used. We assessed the impact of different types of DPP4 inhibitors on PD incidence and stratified the analysis based on age, gender, and diabetic duration. Furthermore, DPP4 inhibitor users were compared with four classes of OADs. All statistical analyses were two-tailed with a significance level of 0.05. STATA 15.1 (STATA Corp, College Station, TX) and SAS 9.4 (SAS Institute Inc., Cary, NC) were used for the analyses.

## Results

### Baseline demographics

**Figure 1:**
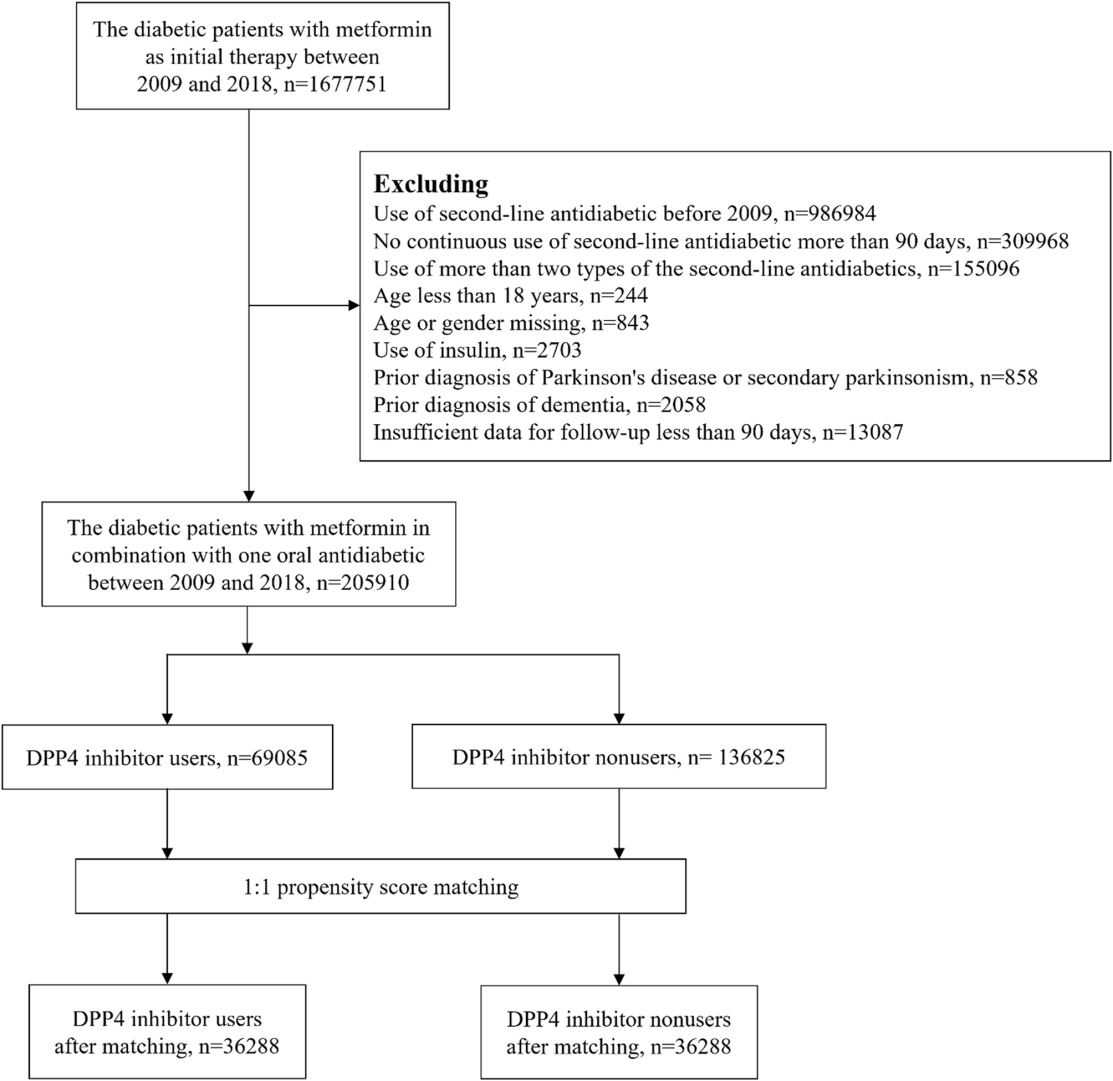
Flow chart of case selection.

A total of 1,677,751 diabetic patients using metformin were included in the analysis for this study. Among these patients, 205,910 individuals met the eligibility criteria, with 69,085 being DPP4 inhibitor users and 136,825 being DPP4 inhibitor nonusers. We employed 1:1 propensity score matching to establish a cohort consisting of 32,688 DPP4 inhibitor users and 32,688 nonusers (Fig. 1). These matched subjects were included in the final analysis.

The demographic characteristics of the study population are summarized in Table 1. The mean follow-up time was 3.7 ± 2.6 years. Over half of the subjects were male, accounting for 52.3% in the matching DPP4 inhibitor user group and 52.2% in the matching DPP4 inhibitor nonuser group. The mean age was 62.3 ± 12.4 years for the DPP4 inhibitor user group and 62.7 ± 12.3 years for DPP4 inhibitor nonuser group. There were no significant differences between the two groups in terms of baseline demographics, such as distribution of age groups and gender, proportion of head injury, and diabetic duration. Conversely, the proportions of hypertension, hyperlipidemia, CHF, cerebrovascular disease, and CKD were higher in the DPP4 inhibitor user group.

**Table 1:** The demographic characteristic by DPP4i user and non-user in metformin cohort.

	Before propensity-score matched				After propensity score propensity-score matched			
	DPP4i user	DPP4i nonuser	SMD	*P* value	DPP4i user	DPP4i nonuser	SMD	*P* value
n	69,085	136,825			36,288	36,288		
Age groups, years old, n (%)				< 0.001				0.29
18–39	2428 (3.5)	5605 (4.1)	0.03		1401 (3.9)	1215 (3.3)	0.028	
40–64	35,092 (50.8)	72,436 (52.9)	0.043		19,309 (53.2)	19,255 (53.1)	0.003	
65–74	18,744 (27.1)	34,509 (25.2)	0.043		9382 (25.9)	9308 (25.7)	0.005	
75+	12,821 (18.6)	24,275 (17.7)	0.021		6196 (17.1)	6510 (17.9)	0.023	
Mean (SD)	63.1 (12.4)	62.3 (12.6)	0.063	< 0.001	62.3 (12.4)	62.7 (12.3)	0.037	< 0.001
Gender, n (%)				< 0.001				0.64
Male	35,359 (51.2)	71,438 (52.2)	0.021		18,995 (52.3)	18,933 (52.2)	0.003	
Female	33,726 (48.8)	65,387 (47.8)	0.021		17,293 (47.7)	17,355 (47.8)	0.003	
Comorbidity, n (%)								
Hypertension	23,605 (34.2)	49,441 (36.1)	0.041	< 0.001	11,221 (30.9)	10,276 (28.3)	0.057	< 0.001
Hyperlipidemia	22,810 (33)	45,252 (33.1)	0.001	0.8	10,875 (30)	9954 (27.4)	0.056	< 0.001
Chronic heart failure	1316 (1.9)	1783 (1.3)	0.048	< 0.001	590 (1.6)	402 (1.1)	0.045	< 0.001
Cerebrovascular disease	3114 (4.5)	5738 (4.2)	0.015	0.001	1420 (3.9)	1255 (3.5)	0.024	0.001
Chronic kidney disease	2662 (3.9)	4761 (3.5)	0.02	< 0.001	1156 (3.2)	1027 (2.8)	0.021	0.005
Obesity	560 (0.8)	860 (0.6)	0.022	< 0.001	227 (0.6)	196 (0.5)	0.011	0.13
Chronic obstructive pulmonary disease	2027 (2.9)	4307 (3.1)	0.012	0.008	964 (2.7)	935 (2.6)	0.005	0.50
Head injury	443 (0.6)	1056 (0.8)	0.016	0.001	205 (0.6)	188 (0.5)	0.006	0.39
Cancer	2314 (3.3)	4118 (3)	0.019	< 0.001	989 (2.7)	917 (2.5)	0.012	0.09
Diabetic duration, years								
Mean (SD)	3.7 (2.7)	3.2 (2.3)	0.221	< 0.001	3.1 (2.3)	3.1 (2.2)	0.006	0.40
Prescription, n (%)								
ACEI/ARB	21,323 (30.9)	39,257 (28.7)	0.048	< 0.001	9704 (26.7)	8964 (24.7)	0.047	< 0.001
CCB	12,833 (18.6)	30,847 (22.5)	0.098	< 0.001	6219 (17.1)	5856 (16.1)	0.027	0.001
Statin	24,053 (34.8)	40,375 (29.5)	0.114	< 0.001	10,718 (29.5)	9878 (27.2)	0.051	< 0.001
NSAID	16,409 (23.8)	38,178 (27.9)	0.095	< 0.001	8291 (22.8)	7494 (20.7)	0.053	< 0.001
Propensity score	0.3517 (0.0758)	0.3274 (0.071)	0.331	< 0.001	0.333 (0.0547)	0.333 (0.0547)	0.001	0.99

*SD* standard deviation, *SMD* standardized mean difference, *DPP4i* dipeptidyl peptidase-4 inhibitor, *ACEI* angiotensin converting enzyme inhibitor, *ARB* angiotensin receptor blocker, *NSAID* non-steroidal anti-inflammatory drugs.

### Incidence of Parkinson's disease among groups

**Figure 2:**
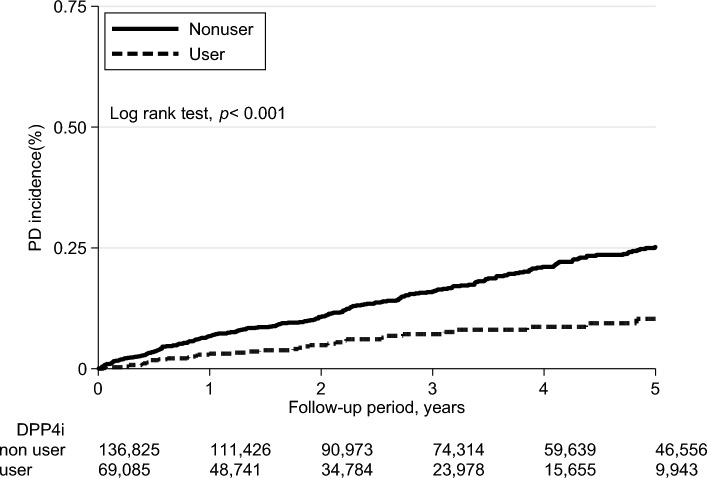
The Parkinson's disease cumulative incidence curve plot by DPP4i user and non-user in metformin cohort.

The overall incidence of PD in this cohort was 0.34 per 1000 person-years. The incidence rate of PD was 0.29 per 1000 person-years in the DPP4 inhibitor user group and 0.55 per 1000 person-years in the DPP4 inhibitor nonuser group. The adjusted hazard ratio (aHR) between the two groups was 0.51 (95% CI 0.39–0.68) (Table 2). The cumulative incidence of PD is illustrated in Fig. 2.

**Table 2:** The risk of Parkinson's disease incidence in DPP4i user and nonuser in metformin cohort by mean DPP4i DDD and DPP4i type.

	Event	Incidence rate (per 1000 person-years)	Model 1	*p* value	Model 2	*p* value	Model 3	*p* value
			Crude hazard ratio (95% CI)^a^		Adjusted hazard ratio (95% CI)^b^		Adjusted hazard ratio (95% CI)^c^	
Parkinson's disease								
DPP4 inhibitors								
Nonuser	105	0.55	Reference		Reference		Reference	
User	44	0.29	0.52 (0.36–0.74)	< 0.001	0.49 (0.38–0.63)	< 0.001	0.51 (0.39–0.68)	< 0.001
Mean DPP4 inhibitors DDD used versus non used^d^								
Lower dose (< 0.32 DDD)	19	0.23	0.40 (0.24–0.65)	< 0.001	0.44 (0.27–0.72)	< 0.001	0.50 (0.35–0.71)	< 0.001
Higher dose (≧ 0.32 DDD)	25	0.38	0.67 (0.43–1.04)	0.07	0.74 (0.48–1.14)	0.017	0.53 (0.36–0.77)	0.001
Types of DPP4 inhibitors^e^								
Sitagliptin	29	0.47	1.10 (0.75–1.61)	0.63	1.22 (0.83–1.80)	0.31	0.96 (0.69–1.32)	0.78
Vildagliptin	11	0.19	0.52 (0.29–0.94)	0.029	0.62 (0.34–1.12)	0.11	0.46 (0.27–0.77)	0.003
Linagliptin	< 4	0.20	0.56 (0.25–1.28)	0.17	0.48 (0.21–1.09)	0.078	0.56 (0.31–1.03)	0.06

*DPP4i* dipeptidyl peptidase-4 inhibitor, *DDD* defined daily dose.

^a^Model 1 was the crude hazard ratio without adjusted other variables.

^b^Adjusted hazard ratio in death competing risk model adjusted age, gender, diabetic duration years, hypertension, hyperlipidemia, chronic kidney disease, obesity, cerebrovascular disease, chronic obstructive pulmonary disease, cancer, head injury, calcium channel blockers, beta-blockers, angiotensin converting enzyme inhibitors or angiotensin receptor blockers, statins, and non-steroidal anti-inflammatory drugs.

^c^Adjusted hazard ratio adjusted the variable in model 2 after propensity score matched and death adjusted competing risk.

^d^The 0.32 was the median of the mean DPP4i DDD dose of DPP4i user and the dose-respond p for the trend after propensity-score matched adjusted model was 0.021.

^e^The DPP4i type of alogliptin and saxagliptin users was excluded from this analysis because there were less case and death < 3.

The median DDD of DPP4 inhibitor in this study was 0.32. In comparison with DPP4 inhibitor nonusers, both the patients with DDD greater than 0.32 and DDD less than 0.32 showed a significant decrease in PD incidence (aHR, 0.50; 95% CI 0.35–0.71 and aHR, 0.53; 95% CI 0.36–0.77, respectively). Among the three specific DPP4 inhibitors analyzed (sitagliptin, vildagliptin, and linagliptin), vildagliptin was associated with a 64% reduction in PD incidence (aHR, 0.46; 95% CI 0.27–0.77), while the other two drugs did not show strong evidence of a lower PD risk (aHR, 0.96; 95% CI 0.69–1.32 and aHR, 0.56; 95% CI 0.31–1.03, respectively).

### Additional analysis

We conducted a stratified analysis to assess the incidence of PD among different age groups, gender, and diabetic duration (Table 3). In the DPP4 inhibitor user group, no PD cases were observed in the individuals younger than 39 years of age. In the remaining age groups, DPP4 inhibitor users had a lower risk of PD compared to nonusers, including those aged 40–64 years (aHR, 0.66; 95% CI 0.33–1.33), 65–74 years (aHR, 0.36; 95% CI 0.22–0.60), and over 75 years (aHR, 0.55; 95% CI 0.39–0.77).

**Table 3:** The Parkinson's disease risk and subgroup analysis in DPP4i user and nonuser in metformin cohort.

	Sample size	DPP4i user	DPP4i nonuser	DPPP4i user versus DPP4i nonuser		
		IR^a^	IR^a^	Adjusted Hazard ratio (95% confidence interval)^b^	*p* value	Subgroup interaction term *p* value
Parkinson's disease	190,920	0.29	0.55	0.49 (0.38–0.63)	< 0.001	
Subgroup						
Age group, years old						
18–39	7912		0.25	-		0.43
40–64	101,823	0.13	0.2	0.66 (0.33–1.33)	0.25	
65–74	48,423	0.43	1.36	0.36 (0.22–0.60)	< 0.001	
75+	32,762	1.61	3.27	0.55 (0.39–0.77)	< 0.001	
Gender						
Male	99,700	0.46	1.00	0.49 (0.34–0.71)	< 0.001	0.83
Female	91,220	0.44	1.03	0.48 (0.33–0.70)	< 0.001	
Diabetic duration, years						
< 3	86,164	0.52	0.91	0.66 (0.46–0.95)	0.027	0.05
≥ 3	104,756	0.4	1.08	0.40 (0.28–0.59)	< 0.001	
Add-on OAD of DPP4i nonuser						
Sulfonylureas	162,591	0.45	1.03	0.48 (0.36–0.62)	< 0.001	
Meglitinides	69,439	0.45	1.79	0.39 (0.25–0.59)	< 0.001	
Alpha-glucosidase inhibitors	72,979	0.45	0.93	0.51 (0.33–0.80)	0.003	
Thiazolidinedione	71,299	0.45	0.44	0.97 (0.50–1.90)	0.94	

*DPP4i* dipeptidyl peptidase-4 inhibitor, *OAD* oral antidiabetic.

^a^*IR* incidence rate (per 1000 person-years).

^b^Adjusted hazard ratio in death competing risk model and adjusted age, gender, diabetic duration years, hypertension, hyperlipidemia, chronic kidney disease, obesity, cerebrovascular disease, chronic obstructive pulmonary disease, cancer, head injury, calcium channel blockers, beta-blockers, angiotensin converting enzyme inhibitors or angiotensin receptor blockers, statins, and non-steroidal anti-inflammatory drugs.

Both male and female DPP4 inhibitor users had a significantly lower risk of PD (aHR, 0.49; 95% CI 0.34–0.71 and aHR, 0.48; 95% CI 0.33–0.70, respectively). Regardless of the duration of diabetes being less than 3 years or 3 years or more, the use of DPP4 inhibitors was associated with a reduced risk of PD. The aHR was 0.66 for diabetic duration < 3 years (95% CI 0.46–0.95) and 0.40 for diabetic duration ≥ 3 years (95% CI 0.28–0.59). Moreover, a longer duration of diabetes was correlated with a lower risk of PD after DPP4 inhibitor treatment (*p* for interaction = 0.05).

We examined the influence of different second-line OAD exposure on the occurrence of PD compared to DPP4 inhibitors. There were 71,299 individuals who used TZD as second-line OAD following metformin. The effect on PD risk reduction was comparable between DPP4 inhibitors and TZDs (aHR, 0.97; 95% CI 0.50–1.90). Compared to either sulfonylureas, meglitinides, or alpha-glucosidase inhibitors, DPP4 inhibitors showed a consistent reduction in the risk of PD (Table 3).

## Discussion

This study assessed the incidence of PD in the Taiwanese population after exposure to DPP4 inhibitors, with a total of 205,910 patients being enrolled. The findings demonstrated a statistically significant decrease in the risk of PD with the use of DPP4 inhibitors. Among the diabetic patients using metformin in combination with a DPP4 inhibitor, the incidence rate was 0.29 per 1000 person-years, compared to 0.55 per 1000 person-years in diabetic patients using metformin plus another add-on OAD. The incidence rate decreased by 55% with exposure to DPP4 inhibitors. After propensity score matching, the PD risk reduction effect of DPP4 inhibitors remained consistent.

The pathogenesis shared by diabetes and neurodegenerative disease involves neuroinflammation, mitochondrial dysfunction, and oxidative stress. Incretin-based therapies provide beneficial effects on neuroprotection by suppressing neuroinflammation, reducing apoptosis, increasing cell viability, restoring neurite outgrowth, decreasing oxidative stress, and strengthening the integrity of the BBB^13,24^. DPP4 inhibitors were demonstrated to have positive effects on the nigrostriatal degeneration in diabetic patients with Parkinson's disease^16^. Several population-based studies have investigated the association between DPP4 inhibitors and the incidence of PD. Two studies by Svenningsson et al. and Brauer et al. found a reduced incidence of PD among patients receiving DPP4 inhibitors, with an adjusted odds ratio of 0.23 (95% CI 0.07–0.074) and an adjusted incidence rate ratio of 0.64 (95% CI 0.43–0.88), respectively^17,18^. In contrast, Rhee et al. reported a significantly increased risk of PD in DPP4 inhibitor-exposed patients compared with non-exposed patients, with an aHR of 1.204 (95% CI 1.104–1.312)^19^. Another case–control population-based study by Sunnarborg et al. did not find a significant effect of DPP4 on the incidence of PD, but the sample size was relatively small. A meta-analysis that pooled data from three population-based studies showed no significant association between the use of DPP4 inhibitors and the risk of PD, with a hazard ratio of 0.69 (95% CI 0.35–1.38; *I*^2^, 88%)^25^. In line with the results of Svenningsson et al. and Brauer et al., our research also found a potential protective effect of DPP4 inhibitor exposure on PD, with an aHR of 0.43 (95% CI 0.25–0.74; *p* = 0.002). Additionally, Brauer et al. reported an incidence rate of 0.5 per 1000 person-year among diabetic individuals exposed to DPP4 inhibitors^18^. In our study, which included diabetic people receiving metformin and one add-on OAD (either a DPP4 inhibitor or another OAD), a lower incidence rate of 0.29 per 1000 person-year was reported.

The practice guideline for T2DM recommends metformin as the initial pharmacological therapy if OAD is indicated. Addition of other antidiabetic agents, such as DPP4 inhibitors, in a stepwise manner is recommended to achieve optimal glycemic control^21^. Both in vivo and in vitro studies have shown that metformin significantly reduces the phosphorylation of alpha-synuclein, inhibits over-activation of microglia, decreases generation of mitochondrial reactive oxygen species, and suppresses the neuroinflammation^26–30^. In term of real-world evidence, the results have been controversial. Two meta-analyses have reported a lack of significant association between metformin use and the risk of PD^25,31^. In this study, we specifically selected patients using metformin as part of their combination therapy to compare the effect of DPP4 inhibitors and other OADs (sulfonylureas, meglitinides, alpha-glucosidase inhibitors, and TZDs). By adopting this approach, we aimed to mitigate the potential confounding effect of metformin and enhance the relevance of the study to the current clinical scenario.

Vildagliptin was significantly correlated with a lower risk of PD (aHR 0.46; 95% CI 0.27–0.77), while sitagliptin and linagliptin only showed a trend. Previous studies have indicated potential antiparkinsonian effects of vildagliptin. In a rat model with high-fat diet consumption, vildagliptin restored the insulin receptor signaling pathway and reduced the concentration of mitochondrial reactive oxygen species production^15^. In a rat rotenone model, vildagliptin enhanced the antioxidant pathway by normalizing the receptor for advanced glycated end products^32^. The sample size of patients using alogliptin or saxagliptin was insufficient for evaluation. The antiparkinsonian impact of each type of DPP4 inhibitor, except for vildagliptin, on real-world practice remains unknown. Given the limited number of cases in this analysis, further studies with a larger sample size are warranted to elucidate the association. We also compared the effectiveness of DPP4 inhibitors in reducing the prevalence of PD among patients with different diabetic durations and different age groups. A higher reduction in PD risk was observed in those with a diabetic duration of more than 3 years, which may be attributed to the longer duration of drug use. Furthermore, a similar trend was observed in patients older than 65 years. However, it should be noted that this finding may be influenced by the lower prevalence of PD in the younger population.

The median DDD of DPP4 inhibitors in our study was 0.32. However, further analysis did not reveal a dose-dependent benefit with different DDD. The group of patients with lower DDD (< 0.32 DDD) showed a 50% decrease in PD risk, while the higher DDD group (≥ 0.32 DDD) showed a 47% reduction. However, as nonadherence to OADs is common in Taiwan^33^, it may impact the accuracy of DDD values, consequently leading to the absence of a dose-dependent effect. Furthermore, we evaluated the influence of different OADs on the risk of PD compared to DPP4 inhibitors. The results did not show significant differences when the patient in the non-DPP4 inhibitor group received combination therapy with metformin plus TZD. TZD, a peroxisome proliferator-activated receptor gamma agonist, shared similarities with DPP4 inhibitors in terms of its proposed anti-neuroinflammatory effects. Several population-based studies have shown the neuroprotective potential of TZD^34–37^, which may contribute to the lack of difference between the DPP4 inhibitor-exposed group and the TZD-exposed group. However, it should be noted that the sample size of patients using TZD was small in this study, which may have affected the comparison of these two medications.

The study has several limitations. First, it relied on retrospective data from the NIHRD. This introduces the possibilities of disease misclassification and medication non-adherence among patients. To ensure the reliability of diagnosis, we referred to a previous study’s definition of PD diagnosis, which required at least one inpatient or outpatient medical claim with a diagnostic code for PD, in addition to the use of antiparkinsonian medications, and reported a positive predictive value of 94.8% for diagnosing PD using ICD codes^38^. In our study, we further restricted the antiparkinsonian medications to levodopa, dopamine agonist, and monoamine oxidase B inhibitor, as these are recommended drugs for symptomatic treatment of early PD^39^. Second, the sample size was limited. The estimated prevalence of PD in the Taiwanese population was between 84.8 and 147.7 per 100,000 individuals^38^. Although the incidence of PD was higher among diabetic population^40^, and a total of 1,677,751 diabetic patients were included in this research, there were only 149 cases of PD. Third, the NHIRD is primarily composed of claim data and does not encompass all aspects of medical records. Certain crucial variables, such as HbA1c values (which may be related to the severity of PD^41^), and lifestyle factors including smoking and coffee consumption (known to be inversely associated with PD^42,43^), are not retrievable from the NHIRD. Forth, given that PD is a chronic condition, the index date may not precisely represent the actual onset date. Extracting data on antidiabetic drugs only one year preceding the PD diagnosis might be insufficient to comprehensively evaluate the relationship between DPP4 inhibitors and the reduced occurrence of PD. However, it is worth emphasizing that the median duration of DPP4 inhibitor usage in our DPP4 inhibitor user cohort was indeed 3.4 years, indicating that these individuals were chronic users. Such an extended duration of drug use can also mitigate concerns about a reverse relationship. Future studies should consider analyzing a more extended period of antidiabetic medication usage to provide a comprehensive understanding. Finally, it should be noted that DPP4 inhibitors were introduced in Taiwan in 2009. The relatively short duration of DPP4 inhibitor administration may have led to insufficient follow-up time to fully clarify the association between this drug and the risk of PD.

In conclusion, our study suggests that the use of DPP4 inhibitors was associated with a reduced risk of developing PD in the Taiwanese diabetic population. Specifically, among the DPP4 inhibitors analyzed in this cohort, there was a significant correlation between vildagliptin use and a lower risk of PD. These findings show that DPP4 inhibitors may have additional neuroprotective effects beyond their role in glycemic control, making them a treatment option for diabetic patients considering the risk of neurodegenerative diseases, such as PD.

### Supplementary Information

Supplementary Information.

## Data Availability

Researchers can apply to access the NHIRD for research purposes by adhering to the application policies of Taiwan's Ministry of Health and Welfare and the NHI Administration. More information can be found at https://nhird.nhri.org.tw/.
