# Supplementary material for: Use of dipeptidyl peptidase-4 inhibitors was associated with a lower risk of Parkinson’s disease in diabetic patients
Source: Sci Rep. 2023 Dec 15;13:22489. doi: 10.1038/s41598-023-49870-z (PMC10728170; doi:10.1038/s41598-023-49870-z)
Supplement: Supplementary file 1 — Supplementary Information. [file 41598_2023_49870_MOESM1_ESM.docx]

**Supplementary Information**

**Supplemental table S1**: **The ICD-9-CM and ICD-10 diagnostic codes of comorbidities**.

| Item | ICD-9-CM | ICD-10 |
| --- | --- | --- |
| **Inclusion criteria** |  |  |
| Parkinson's disease | 332.0 | G20 |
| Type 2 diabetes | 250.x | E11 |
| **Exclusion criteria** |  |  |
| Secondary parkinsonism | 332.1 | G21.11, G21,19, G21.8 |
| Type 1 diabetes | 250.x | E10 |
| Dementia | 290, 1851.2, 294.1, 331.0, 331.1, 331.2 | F05, F03.9, G03.9, G31.1, F01.5, F01.51, F02.81, G31.01, G31.09, G31.83, I69.91 |
| **Comorbidities** |  |  |
| Hypertension | 401–405 | I10-I16 |
| Diabetic | 250.x | E10, E11 |
| Hyperlipidemia | 272.x (excluding 272.5x, 272.8) | E78 |
| Cardiovascular disease | 410, 411 | I20-I22 |
|  |  |  |
| Congestive heart failure | 428 | I50 |
| Stroke | 430.x-438.x | G45-G46, I60-I66, I68, I69 |
| Liver cirrhosis (Non-alcoholic) | 571.5, 571.6 | K74.0, KJ74.1, K74.2, K74.60, K74.69, K74.3, K74.4, K74.5 |
| Obesity | 278 | E65, E66, E67, E68 |
| Chronic obstructive pulmonary disease | 491, 492, 496 | J40-J44 |
|  |  |  |
| Hepatitis B virus infection (HBV) | 070.2, 070.3, V02.61 | B18.0, B18.1, B19.1 |
| Chronic kidney disease | 016.0, 095.4, 189.0, 189.9, 223.0, 236.91, 250.4, 271.4, 274.1, 283.11, 403.01, 403.11, 403.91, 404.02, 404.12, 404.92, 404.03, 404.13, 404.93, 440.1, 442.1, 447.3, 572.4, 580, 580.0, 580.4, 580.8, 580.81, 580.89, 580.9, 581, 581.0, 581.1, 581.2, 581.3, 581.8, 581.81, 581.89, 581.9, 582, 582.0, 582.1, 582.2, 582.4, 582.8, 582.81, 582.89, 582.9, 583, 583.0, 583.1, 583.2, 583.4, 583.6, 583.7, 583.8, 583.81, 583.89, 583.9, 584, 584.5, 584.6, 584.7, 584.8, 584.9, 585, 586, 587, 588, 588.8, 588.9, 591, 642.1, 646.2, | I12, I13, N00, N01, N02, N03, N04, N05, N07, N11, N14, N17, N18, N19, Q61 |

**Supplemental table S2**: **The ATC code of prescription**.

| Prescription | ATC code |
| --- | --- |
| Angiotensin converting enzyme inhibitors/angiotensin receptor blockers | C09, C10BX |
| Beta blockers | C03C07 (excluding C07AA05), C09BX, C09DX |
| Calcium channel blockers | C08, C08G, C09BB, C07FB, C10BX |
| Diuretics | C03, C07B, C07C, C07D, C08G, C09BA, C09DA |
| Statins | C10AA, C10B |
| Non-steroidal anti-inflammatory drugs | M01A |
| Metformin | A10BA, A10BD01, A10BD02, A10BD03, A10BD05, A10BD07, A10BD08, A10BD10, A10BD11 |
| Sulfonylureas | A10BB, A10BC, A10BD01, A10BD02, A10BD04, A10BD06 |
| Meglitinides | A10BX02, A10BX03, A10BX08 |
| Alpha-glucosidase inhibitors | A10BF |
| Thiazolidinediones | A10BG, A10BD03, A10BD04, A10BD05, A10BD06, A10BD09 |
| Dipeptidyl peptidase-4 inhibitors | A10BH, A10BD07, A10BD08, A10BD09, A10BD10, A10BD11 |
| Sitagliptin | A10BH01, A10BH07 |
| Vildagliptin | A10BH02, A10BH08 |
| Saxagliptin | A10BH03, A10BH10 |
| Linagliptin | A10BH05 |
| Alogliptin | A10BH04, A10BH09 |
| Insulin | A10A |
| Hepatitis B virus treatment | J05AF05, J05AF07, J05AF08, J05AF10, J05AF11 |
| Hepatitis C virus treatment | L03AB |
